# NullHap – a versatile application to estimate haplotype frequencies from unphased genotypes in the presence of null alleles

**DOI:** 10.1186/1471-2105-9-330

**Published:** 2008-08-05

**Authors:** Robert M Nowak, Rafał Płoski

**Affiliations:** 1 Department of Electronics and Information Technology, Institute of Electronic Systems, Warsaw University of Technology, Warsaw, Poland; 2 Department of Medical Genetics, Medical University of Warsaw, Warsaw, Poland

## Abstract

**Background:**

Laboratory techniques used to determine haplotypes are often too expensive for large-scale studies and lack of phase information is commonly overcome using likelihood-based calculations. Whereas a number of programs are available for that purpose, none of them can handle loci with both multiple and null alleles.

**Results:**

Here we present a description of a modified Expectation – Maximization algorithm as well as its implementation (NullHap) which allow to effectively overcome these limitations. As an example of application we used Nullhap to reanalyze published data on distribution of *KIR *genotypes in Polish psoriasis patients and controls showing that the *KIR2DS4/1D *locus may be a marker of *KIR2DS1 *haplotypes with different effects on disease susceptibility.

**Conclusion:**

The developed application can estimate haplotype frequencies for every type of polymorphism and can effectively be used in genetic research as illustrated by a novel finding regarding the genetic susceptibility to psoriasis.

## Background

Laboratory techniques used to determine haplotypes [1] are often too expensive for large-scale studies. The lack of phase information provided by the popular typing methods could be overcome using likelihood-based calculations [2], which estimate haplotype frequencies in a population, and reconstruct the haplotype pair in each individual. This approach is more cost-effective and powerful than linkage analysis [3], and gives more information than single marker-based methods [4].

Haplotype estimation procedures typically use maximum likelihood approach. The most popular algorithm implemented for example in Arlequin [5] is The Expectation – Maximization algorithm (EM) [6] but other methods were also proposed: Bayesian method using a pseudo-Gibbs sampler [7], partition-ligation [8], Monte Carlo [9] and Hidden Markov Model [10].

A frequent shortage of available software packages [5,7] is the lack of possibility to analyze loci where null variants occur with an appreciable frequency. In a diploid organism, a null allele is a variant which is not detected in genotyping, because of a deletion of an entire locus or because of a mutation interfering with analysis. This makes it impossible to distinguish between some heterozygous and homozygous genotypes [11]. For example, if there is only one alternative allele *A*_1 _besides the null allele *A*_0_, then there are three possible haplotype pairs: *A*_1_/*A*_1_, *A*_1_/*A*_0 _and *A*_0_/*A*_0_, but only two kinds of experimental observations: *A*_0 _and *A*_1_. An example of a genetic system, which is at present intensely studied [11] and which contains null alleles, is the locus encoding killer immunoglobulin-like receptors (*KIR*) of natural killer (NK) cells.

To our knowledge, the only available computer program designed to handle null alleles is Haplo-IHP [12], which, however, has a shortcoming of being applicable only to biallelic loci. The purpose of our work was to design a versatile application for estimation of haplotypes from unphased population data useful for multiallelic polymorphism with and/or without null alleles.

## Implementation

The null variants decrease the number of different genotypes *G *which can be observed, equation (1), when the *k *polymorphic loci are analyzed and each locus has *l*_*i *_different variants (optionally including a null variant) for *i*-th locus, *δ*_*i *_= 1 if *i*-th locus has null allele, otherwise *δ*_*i *_= 0. The number of haplotypes is $H=\prod_{i=1}^{k}l_{i}$.

(1) $$G=\prod_{i=1}^{k}\frac{(l_{i}-\delta_{i})(l_{i}+1-\delta_{i})+2\delta_{i}}{2}$$

The average number of haplotype resolutions which give genotype *j *(when phase information is lost) grows exponentially with the number of observed loci, thus full space search algorithm cannot be used to find the best haplotype frequencies. The equation (2) provides the number of haplotype resolutions *r*_*j *_which give genotype *j*, where *s*_*j *_is the number of observed heterozygous and *t*_*j *_is the number of observed (not null) alleles for loci with null allele(s).

(2) $$r_{j}=\{\begin{array}{ll} 2^{s_{j}-1}3^{t_{j}} & \text{for }s_{j}>0\\ \frac{3^{t_{j}}+1}{2} & \text{for }s_{j}=0 \end{array}$$

### Maximum likelihood approach to estimate haplotypes

In the maximum likelihood approach haplotype frequencies *h*_*i *_are estimated to maximize the probability of the given sample of genotyping data. The sample of genotyping data from *n *individuals is simplified to a vector *S *= (*n*_1_, *n*_2_,..., *n*_*G*_), where *G *is the number of different genotyping data (with a lack of phase information, equation (1)), and *n*_*j *_is the number of individuals having *j*-th genotype, $\sum_{j=0}^{G}n_{j}=n$.

The conditional probability of sample *S*, given each genotype probability *g*_*i*_, and assuming unrelatedness of individuals in the sample is provided in equation (3), where *α *does not depend on *g*_*j*_.

(3) $$P(S|g_{1},g_{2},...,g_{G})=\frac{n!}{n_{1}!n_{2}!...n_{G}!}\prod_{j=1}^{G}g_{j}^{n_{j}}=\alpha \prod_{j=1}^{G}g_{j}^{n_{j}}$$

The frequency of genotype *g*_*j *_is the sum of frequencies of respective haplotype pairs *z*_*mn*_, and with Hardy-Weinberg equilibrium (HWE) assumption, it is calculated from haplotype frequencies as shown in equation (4), where *z*_*mn *_is the frequency of haplotype pair *m *and *n*, *r*_*j *_is the number of haplotype pairs for the *j*-th genotype (equation 2), and *h*_*m*_, *h*_*n *_are the frequencies of haplotypes *m *and *n *respectively.

(4) $$\begin{array}{ccc} g_{j}=\sum_{i=0}^{r_{j}}z_{mn}, & \text{where} & z_{mn}=\{\begin{array}{ll} h_{m}^{2} & \text{for }m=n\\ 2h_{m}h_{n} & \text{for }m\neq n \end{array} \end{array}$$

The estimation of haplotype frequencies to maximize the probability of the observed sample can be described as optimization, the equation (5) summarizes the considered approach.

(5) $$\begin{array}{c} \underset{h_{1},h_{2},...,h_{H}}{\arg\max} \end{array}P(S|h_{1},h_{2},...,h_{H})=\underset{h_{1},h_{2},...,h_{H}}{\arg\max}\prod_{j=1}^{G}(\sum_{i=0}^{r_{j}}z_{mn}{)}^{n_{j}}$$

### Extended EM algorithm

The EM alternates between performing an expectation step *E*^(*t*)^, which computes an expectation value of unknown parameters, here the frequencies of haplotype pairs, and a maximization step *M*^(*t*)^, which computes the value of parameters by maximizing the probability of observed data. The parameters found on the *M*^(*t*) ^step are then used to begin another *E*^(*t*+1) ^step, and the process is repeated until the parameters are changed.

The description of algorithm details for the observed genotype data of *k *linked loci, *l*_*i *_variants for *i*-th locus, and the sample *S *= (*n*_1_, *n*_2_,..., *n*_*G*_) is given below.

#### Initiation

The EM algorithm could be trapped into a local maximum, therefore multiple random starts are employed (any number determined by the user) in order to help the algorithm reach the global maximum. If *n *> 1 starting points are specified, for *i-th *point, the program calculates the mean error between the first and *i-th *estimate, and if this exceeds a predefined value (default = 0.05) a message is displayed about possible multiple local maxima. Since this feature increases computational time, it is optional.

If no random starts are used, the initial haplotype pair frequencies are set as described in equation (6) (the *E*^0 ^step). For each haplotype resolution, the initial frequency depends only on the number of haplotype pairs for the given genotype. A similar initiation is described in [6].

(6) $$z_{mn}^{\left(0\right)}=\frac{1}{r_{j}}\text{where the }mn\text{ gives the }j\text{ genotype}$$

#### Maximization step

In this step, the typical EM algorithm was adopted, the only modification consisting of the fact that the genotype frequency calculation was performed as a sum of corresponding haplotype pair frequencies, equation (4), taking into account that the heterozygotes with null allele are genotyped identically as homozygotes without null allele.

Next, the haplotype pair frequencies are corrected, to maximize the probability of a given sample. Details are given in equation (7), where $z_{mn}^{\left(t\right)}$ is the input haplotype pair frequency, $g_{j}^{\left(t\right)}$ is the calculated genotype frequency (inclusive of appropriate heterozygous genotypes with null variants), $z_{mn}^{\left(t+1\right)}$ is the output haplotype pair frequency, corrected to maximize the observed sample, *n*_*j *_is the number of observed genotypes *g*_*j *_in sample and *n *is the number individuals in the sample.

(7) $$z_{mn}^{\left(t+1\right)}=\frac{n_{j}}{n}*\frac{z_{mn}^{\left(t\right)}}{g_{j}^{\left(t\right)}}$$

#### Expectation step

Haplotype frequencies *h*_*m*_s are calculated from the given haplotype pair frequencies *z*_*mn*_s, as a half of the sum of frequencies of all pairs of haplotypes in which given haplotype occurs. The next expected haplotype pair frequencies are calculated using haplotype frequencies as described in equation (8).

(8) $$\begin{array}{cc} z_{mn}^{\left(t+1\right)}=\{\begin{array}{cc} {\left(h_{m}^{\left(t\right)}\right)}^{2} & \text{for }m=n\\ 2h_{m}^{\left(t\right)}h_{n}^{\left(t\right)} & \text{for }m\neq n \end{array} & h_{m}^{\left(t\right)}=\frac{1}{2}(\sum_{i}z_{im}^{\left(t\right)}+\sum_{j}z_{mj}^{\left(t\right)}) \end{array}$$

#### Stop conditions

The algorithm stops, when the stability of estimations between the following steps is obtained, i.e. the absolute difference between the calculated frequencies is less then *ε *(equation 9). The default threshold value for *epsilon *is 10^-5^, and can be changed by a program option.

(9) $$\sum_{i=1}^{R}\left|z_{i}^{\left(t+1\right)}-z_{i}^{\left(t\right)}\right|<\varepsilon$$

The final step is calculation of the haplotype frequencies (another E step), and of the conditional probability of the haplotype pair, given genotype estimation (equation 10).

(10) $$z_{mn}|g_{j}=\frac{z_{mn}}{g_{j}}=\frac{z_{mn}}{\sum_{x}^{r_{j}}z_{x}}$$

## Results and Discussion

The described algorithm was implemented using C++ and the Boost libraries [13] and called NullHap. The main advantage of our application is the ability to handle problems, when one or more multiallelic loci containing null variant occur.

NullHap was tested on simulated and real data sets and its performance was compared with those of previously described programs: Arlequin [5], PHASE [7] and Haplo-IHP [12].

### Test on generated data sets

Firstly, the simulated data sets were obtained as the most probable samples generated for polymorphisms with varying locus characteristics, and accuracy of estimated frequencies for different computer programs was analyzed. An example of assumed and estimated frequencies used in one such simulation is shown in Table 1. In Table 2, results of six simulations are summarized by giving a mean absolute percentage error, calculated as shown in equation (11), where *x *is the assumed frequency, and x* is the calculated one.

**Table 1:** Assumed and estimated haplotype frequencies

haplotype	frequency *h*_*i*_				
	assumed	Arlequin	PHASE	Haplo-IHP	NullHap
*A*_0_*B*_1_*C*_0_	0.2	0.068	0.068	0.294	0.20
*A*_0_*B*_1_*C*_1_	0.2	0.172	0.172	0.294	0.20
*A*_0_*B*_2_*C*_0_	0.1	0.034	0.034	0.147	0.10
*A*_0_*B*_2_*C*_1_	0.02	0.038	0.038	0.029	0.02
*A*_0_*B*_3_*C*_0_	0.02	0.007	0.007	0.0	0.02
*A*_0_*B*_3_*C*_1_	0.02	0.017	0.017	0.0	0.02
*A*_0_*B*_4_*C*_0_	0.02	0.007	0.007	0.0	0.02
*A*_0_*B*_4_*C*_1_	0.02	0.017	0.017	0.0	0.02
*A*_1_*B*_1_*C*_0_	0.1	0.089	0.089	0.147	0.10
*A*_1_*B*_1_*C*_1_	0.02	0.125	0.125	0.029	0.02
*A*_1_*B*_2_*C*_0_	0.02	0.028	0.028	0.029	0.02
*A*_1_*B*_2_*C*_1_	0.02	0.042	0.042	0.029	0.02
*A*_1_*B*_3_*C*_0_	0.02	0.015	0.015	0.0	0.02
*A*_1_*B*_3_*C*_1_	0.02	0.035	0.035	0.0	0.02
*A*_1_*B*_4_*C*_0_	0.02	0.015	0.015	0.0	0.02
*A*_1_*B*_4_*C*_1_	0.02	0.035	0.035	0.0	0.02
*A*_2_*B*_1_*C*_0_	0.02	0.028	0.029	0.0	0.02
*A*_2_*B*_1_*C*_1_	0.02	0.078	0.078	0.0	0.02
*A*_2_*B*_2_*C*_0_	0.02	0.019	0.019	0.0	0.02
*A*_2_*B*_2_*C*_1_	0.02	0.039	0.039	0.0	0.02
*A*_2_*B*_3_*C*_0_	0.02	0.013	0.013	0.0	0.02
*A*_2_*B*_3_*C*_1_	0.02	0.033	0.033	0.0	0.02
*A*_2_*B*_4_*C*_0_	0.02	0.013	0.013	0.0	0.02
*A*_2_*B*_4_*C*_1_	0.02	0.033	0.033	0.0	0.02

error	-	79%	79%	82%	0%

The assumed and estimated haplotype frequencies for a polymorphism with 3 loci: A(multiallelic with null variant), B(multiallelic), C(biallelic with null variant).

**Table 2:** Haplotype estimation frequency error

No	example description	error			
		Arlequin	PHASE	Haplo-IHP	NullHap
1	biallelic loci: A(*A*_1_, *A*_2_), B(*B*_1_, *B*_2_), C(*C*_1_, *C*_2_) no null variants	0%	0%	0%	0%
2	biallelic loci: A(*A*_0_, *A*_1_), B(*B*_0_,*B*_1_), C(*C*_0_, *C*_1_), null variants: *A*_0_, *B*_0 _and *C*_0_	61%	50%	1%	0%
3	multiallelic loci: A(*A*_1_, *A*_2_, *A*_3_), B(*B*_1_, *B*_2_, *B*_3_), no null variants	0%	1%	78%	0%
4	multiallelic loci: A(*A*_0_, *A*_1_, *A*_2_), B(*B*_0_,*B*_1_, *B*_2_), null variants: *A*_0 _and *B*_0_	62%	62%	100%	0%
5	multiallelic and biallelic loci with null variants: A(*A*_0_,*A*_1_,*A*_2_), B(*B*_0_,*B*_1_), C(*C*_0_,*C*_1_)	62%	48%	64%	0%
6	details in Table 2, A(*A*_0_,*A*_1_,*A*_2_), B(*B*_1_,*B*_2_,*B*_3_,*B*_4_), C(*C*_0_,*C*_1_)	79%	79%	82%	0%

Haplotype estimation frequency error for six polymorphisms with varying locus characteristics.

(11) $$error=\frac{1}{N}\sum_{i=1}^{N}\left|\frac{x-x^{*}}{x}\right|$$

Since it may not be known beforehand, whether a locus has a null allele, we also checked performance of NullHap which was run assuming the presence of a null allele in each locus. Such an approach allows to screen the likelihood of the presence of a null allele in a given locus by evaluating the frequencies of haplotypes containing this allele. An appreciable frequency of any such haplotype in the output indicates the need to include a null allele in this particular locus. Otherwise, the conclusion is, that given locus most likely does not contain a null variant. Alternatively, genotypes of each locus could be analyzed for deviation from HWE by any of the available programs. When typing mistakes are excluded, deviation from HWE strongly indicates the presence of a null allele.

**Figure 1:**
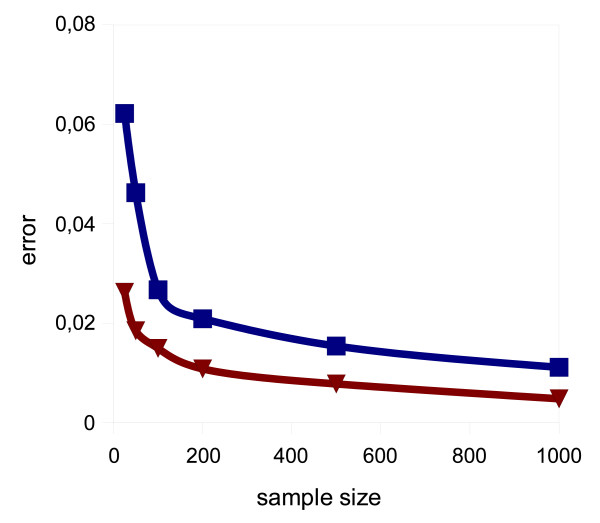
**Effect of sample size on accuracy of estimation**. Effect of sample size on accuracy of estimation of haplotype frequencies. Ten samples of 25, 50, 100, 200, 500, 1000 individuals were generated from population in HWE. The error in function of sample size is shown. The haplotype distribution is given in example 5 (red) and example 6 (blue) in Table 2, respectively.

Secondly, the effect of sample size on the performance of the method was investigated. This was done by generating *k *random samples of 25, 50, 100, 200, 500 and 1000 individuals from an infinite population in HWE. The haplotype frequencies were estimated and median of *k *mean absolute errors (calculated as $error=\frac{1}{N}\sum_{i=1}^{N}\left|x-x^{*}\right|$, where *N *is the number of individuals in the sample) was calculated. The results obtained for haplotype distributions such as those given in examples 5 and 6 in Table 2 are illustrated in Figure 1. As can be seen, with a sample size of 200 individuals, an error of approximately 2% can be expected in haplotype frequency estimation, whereas a lower sample size may lead to substantially higher errors.

Thirdly, tests of the effect of different levels of HWE violation on the accuracy of the algorithm were performed. The degree of HWE violation was modeled by increasing values of inbreeding coefficient *f *as defined by Weir [14,15], equation (12).

(12) $$z_{mn}=\{\begin{array}{ll} h_{m}^{2}(1-f)+h_{m}f & \text{for }m=n\\ 2h_{m}h_{n}(1-f) & \text{for }m\neq n \end{array}$$

**Figure 2:**
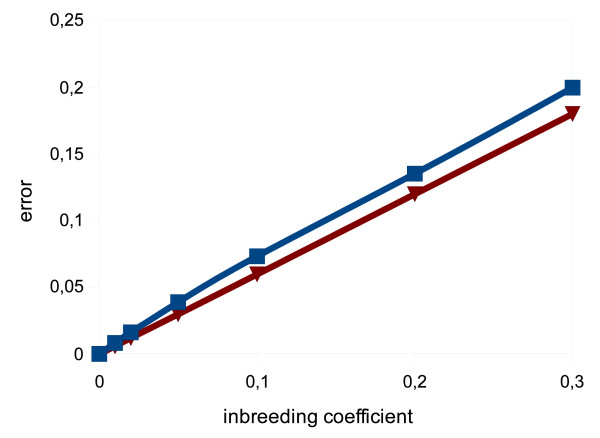
**Effect of HWE violation on accuracy of estimation**. Effect of HWE violation on the accuracy of the algorithm. The figure shows the error in function of inbreeding coefficient *f *for two polymorphisms characterized in Table 2 (example 5 – red line, example 6 – blue line).

As can be seen from Figure 2, there was a linear correlation of inbreeding coefficient *f *with the accuracy of estimation of haplotype frequencies.

**Figure 3:**
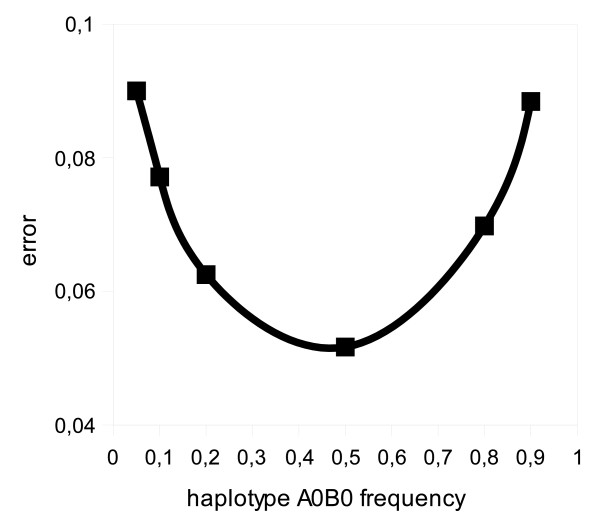
**Effect of haplotype frequency on the error of the estimation**. Effect of haplotype frequency on the error of the estimation. Ten samples of 1000 individuals were generated for population in HWE, for a 2 locus polymorphism: A with variants *A*_0_, *A*_1_, *A*_2 _and B with variants *B*_0_, *B*_1_. The graph shows the error of haplotype frequency estimation in function of assumed frequency of this haplotype.

Finally, to evaluate the effect of haplotype frequency on the error of the estimation, 10 samples of 1000 individuals were generated from a population in HWE, for a simple two loci polymorphism: *A *with variants *A*_0_, *A*_1_, *A*_2 _and *B *with variants *B*_0_, *B*_1_. The frequencies of haplotypes *A*_0_*B*_1_, *A*_1_*B*_0_, *A*_1_*B*_1_, *A*_2_*B*_0_, *A*_2_*B*_1 _were fixed and equal to 0.19, 0.18, 0.16, 0.1, 0.04 and 0.02 respectively, whereas the frequency of haplotype *A*_0_*B*_0 _varied from 0.05 to 0.9. Results expressed as median of mean absolute percentage error (equation (11)) are shown in Figure 3. As can be seen, the lowest error occured with haplotype frequency close to 0.5.

### Performance tests

We also performed analysis of computational time in different scenarios. Results presented for appropriate applications are shown in Table 3. All computations were achieved on Celeron M 1.6 GHz, 1 GB RAM, under Debian Linux or Windows XP.

**Table 3:** Computational time comparison

loci	number of haplotypes	observ.	null alleles	time for application			
				Arlequin	Phase	HaploIHP	NullHap
2	6	100	no	0.13 s	46 s	0.5 s	0.07 s
2	9	100	no	0.06 s	47 s	0.15 s	0.04 s
3	8	100	no	0.04 s	69 s	0.58 s	0.02 s
2	504	99	no	0.22 s	53 s	-	37 s
2	540	99	no	0.34 s	58 s	-	39 s
5	32	200	yes	-	-	14 s	0.78 s
7	128	200	yes	-	-	145 s	13 s
8	256	200	yes	-	-	450 s	61 s
9	512	200	yes	-	-	1300 s(8 s)	209 s
10	1024	200	yes	-	-	3 h (8 s)	2300 s
11	2048	200	yes	-	-	24 h (10 s)	3 h
15	32768	100	yes	-	-	-	48 h

Computational time for considered applications (HaploIHP in parenthesis with greedy algorithm). Results presented only for applications able to handle the given polymorphism, otherwise '-'.

Because the number of haplotypes grows exponentially with the number of considered loci, there is a practical restriction to approximately 50,000 haplotypes, e.g. 15 biallelic loci. We noted with moderate number of loci the restriction is due to computational time, whereas for the very large number of loci (e.g. 100 loci) the memory becomes a limiting factor.

### Tests on real data sets

To perform a test on real data, we first used *HLA-DRB1 *and *HLA-DQB1 *allele distributions among 99 Poles as supplied by [5]. Both loci are multiallelic (36 and 14 variants, respectively) without null variants. The difference between estimated frequencies among programs Arlequin, PHASE and NullHap (i.e. programs handling such loci) was less than 2%.

To test the application in the presence of biallelic loci with null variants, the *KIR *genotypes for 200 Irish subjects [12] were analyzed with NullHap and Haplo-IHP (the only available program suitable for such loci). The difference of estimated frequencies between programs was about 3%.

### Reanalysis of published data indicates that the KIR2DS4/1D locus may be a marker of KIR2DS1 haplotypes with different effects on psoriasis susceptibility

In order to apply NullHap to real data from an association study we reanalyzed the results of Luszczek et al. on distribution of *KIR *genotypes in Polish psoriasis patients and controls [16]. In the original report these authors described an association between *KIR2DS1 *and psoriasis, which was also observed in two subsequent studies from Japan and the US [17,18], but not in a study a of Chinese population [19]. Further analysis of genotype data of Luszczek et al. [16] indicated a role for *KIR *gene variants other than *KIR2DS1 *in conferring susceptibility to psoriasis, suggesting, that distinct *KIR *haplotypes could be responsible for observed associations [20].

The distributions of *KIR *haplotypes among patients and controls obtained with NullHap are given in Table 4. Because the structure of the *KIR *region is very complex, it is not fully known which genes are truly allelic, i.e. occupy precisely the same chromosomal locus. At first, in our analysis, the *K2DL2/KIR2DL3*, *KIRDS4/KIR1D*, and *KIR2DS3/KIR2DS5 *genes were treated as alleles. Since in the case of *KIR2DS3 *and *KIR2DS5 *this may be controversial due to some haplotypes which harbor both genes in cis [21], we also repeated the analysis after exclusion of these variants. In all loci a null allele was allowed [21].

**Table 4:** The distribution of KIR haplotypes

Haplo-type #	*KIR 2*						Psoriatis N = 116 (%)	Controls N = 123 (%)	OR	P value*
	DS2	DL2/3	DS3/5	DL1	DS1	DS4/1D				
1	null	3	null	1	1	1D	20 (17)	0	52.5	0.00018
2	1	2	3	null	1	1D	11 (9.6)	0	27	0.0058
3	null	3	3	null	null	1D	6 (5.3)	2 (1.5)	2.9	NS
4	null	3	5	1	1	null	6 (5.2)	7 (5.6)	0.9	NS
5	null	3	3	1	null	1D	15 (13)	30 (24)	0.5	NS
6	1	2	5	null	1	null	3 (2.5)	7 (6.4)	0.5	NS
7	null	3	null	1	null	1D	0	16 (13)	0.03	0.00018
8	1	2	-	null	1	1D	17 (15)	0	43.4	0.00018
9	null	3	-	1	1	1D	16 (14)	0	40.6	0.00018
10	null	3	-	1	1	DS4	6 (5.3)	0	14.5	NS
11	1	2	-	1	null	1D	7 (6)	3 (2.3)	2.4	NS
12	null	3	-	1	1	null	19 (16)	14 (11)	1.6	NS
13	null	3	-	null	null	1D	6 (5)	7 (5.7)	0.9	NS
14	null	3	-	1	null	1D	19 (16)	44 (36)	0.4	NS
15	1	2	-	null	null	DS4	3 (2.4)	8 (6.6)	0.4	NS
16	1	2	-	null	null	1D	0	7 (5.6)	0.07	NS
17	1	2	-	1	1	null	0	7 (5.6)	0.07	NS
18	null	3	-	1	null	DS4	0	7 (5.6)	0.07	NS

*with Bonferroni correction (correction factor = 18)

The distribution of *KIR *haplotypes among psoriasis patients and controls obtained with NullHap based on genotypes reported by Luszczek et al. [16]. Only haplotypes with frequency > 5% in either group are shown. Odds ratio (OR) calculated according to Haldane [22], P value calculated by Fisher exact test. NS -not significant.

As can be seen from Table 4, two haplotypes (#1, #2) were strongly overrepresented among the patients. The fact that these haplotypes encoded *KIR2DS1 *is consistent with the association between this gene and psoriasis [16-18] whereas the lower OR associated with haplotype #2 vs. #1 (27 vs. 52.5) supports the protective effect of *KIR2DS3 *suggested previously [20]. In contrast to haplotypes #1 and #2 other haplotypes encoding *KIR2DS1 *(#4, #6) were not overrepresented among the patients. Both haplotypes encoded *KIR2DS5 *which could be interpreted as the postulated protective effect of this variant [20]. However, whereas the presence of *KIR2DS5 *or *KIR2D3 *offers one explanation of the heterogeneity of the effects of *KIR2DS1 *haplotypes, the inspection of Table 4 shows that the risk – conferring and neutral *KIR2DS1 *haplotypes are also distinguished by the *KIR2DS4/1D *locus, which is a novel observation. As can be seen, the haplotypes #1, #2 share the *1D *variant, whereas the haplotypes #4, #6 both have the *KIR2DS4 *null allele. These effects of *KIR2DS4/1D *locus were also apparent in analysis performed after the exclusion of *KIR2DS3 *and *KIR2DS5 *genes (haplotypes #8 and #9 vs. haplotypes #12 and #17, Table 4).

The fact that *KIR2DS4/1D *and *KIR2DS1 *loci are physically adjacent [21] suggests that the strong predictive effect of their haplotypic combinations may be caused by linkage disequilibrium with an unknown variant in the region, which is primarily associated with psoriasis. The indirect association is particularly plausible for *KIR2DS4/1D *because *KIR 1D *and *KIRDS4 *null (which mark *KIR2DS1 *haplotypes with distinct effects on disease susceptibility) are both non functional and thus should be equivalent physiologically [21]. In case of the *KIR2DS1 *it would be tempting to speculate that the susceptibility conferring effect is limited to a rare allele (absent in controls) being in strong linkage disequilibrium with *1D*. Interestingly, such a theory could explain a lack of association between *KIRDS1 *and psoriasis recently reported in a Chinese population [19].

## Conclusion

The developed application can effectively estimate haplotype frequencies with a performance that is similar or better than those of other available computer programs. It should be emphasized, that the main advantage of the created application is the ability to estimate haplotypes for every type of polymorphism, in particular polymorphisms with multiallelic loci with null variants.

The presented application is under development, and some improvements are planned, such as an additional step removing unimportant haplotypes or the partition-ligation algorithm [8] to speed-up computations for a large number of loci. Other planned improvements are a graphical user interface as well as an import/export module for popular data formats. The new versions will be available at project homepage.

## Availability and requirements

**Project name**: NullHap

**Project homepage**: 

**Operating systems(s)**: OS Portable

**Precompiled binaries**: Windows NT/2000/XP, Debian Linux

**Programming language**: C++

**License**: GNU LGPL

## Authors' contributions

RN adopted algorithm, implemented application, performed calculations and drafted manuscript. RP proposed the idea to develop the software, outlined its main features, participated in validation process and provided biological interpretation of results of reanalysis of KIR haplotypes. All authors read and approved the final manuscript.
